# Effect of food intake on myocardial performance index

**DOI:** 10.1186/s12947-017-0101-z

**Published:** 2017-04-05

**Authors:** Ylva Gårdinger, Anna Dieden, Joanna Hlebowicz, Ola Björgell, Magnus Dencker

**Affiliations:** 1 Department of Translational medicine, Unit of Clinical Physiology and Nuclear Medicine, Skåne University Hospital, Lund University, Malmö, Sweden; 2 Department of Translational medicine, Unit of Radiology, Skåne University Hospital, Lund University, Malmö, Sweden; 3 Department of Clinical Sciences, Division of Medicine, Skåne University Hospital, Lund University, Malmö, Sweden

**Keywords:** Food intake, Echocardiography, Myocardial performance index

## Abstract

**Background:**

Myocardial performance index (MPI) has been investigated in a variety of populations, but the effect of food intake has not been evaluated. We assessed whether myocardial performance index is affected by food intake in healthy subjects.

**Methods:**

Twenty-three healthy subjects aged 25.6 ± 4.5 years were investigated. MPI was measured before, 30 min after, and 110 min after a standardized meal.

**Results:**

MPI decreased significantly (*P* < 0.05) from fasting values 30 min after the meal, and had almost returned to baseline after 110 min. MPI decreased from 0.28 ± 0.06 (fasting) to 0.20 ± 0.07 30 min after eating. At 110 min after eating the index value was almost back to the baseline value 0.26 ± 0.06. (*P* = 0.15).

**Conclusions:**

This study shows that myocardial performance index is affected by food intake in healthy subjects.

## Background

**Fig. 1:**
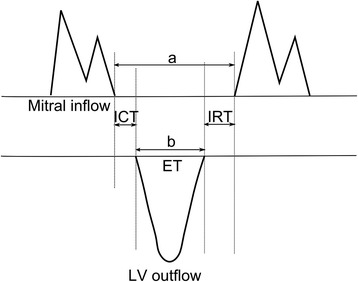
Schematic figure of measurement and calculation of myocardial performance index

Myocardial performance index (MPI), also known as Tei-index, was introduced by Tei in 1995 as a Doppler-derived index of combined systolic and diastolic myocardial performance [1]. MPI is defined as the sum of isovolumetric contraction time (ICT) and isovolumetric relaxation time (IRT) divided by ejection time (ET) (Fig. 1). MPI has been examined in many studies in a variety of populations over the last two decades, and has been used as a measure of total left ventricular function. It has been used for prognostic examinations of patients regarding heart failure [2], acute myocardial infarction [3] and heart transplants [4], as well as in healthy athletes [5] and in yoga practitioners with heart failure [6]. MPI has also been used in several studies regarding pediatric patients; for example helping to detect acute cellular rejection after heart transplant [7], for early detection of chemo-induced cardiotoxicity [8], in investigations concerning cardiovascular changes in children with bicuspid aortic valves [9], and for assessment of ventricular function in obese children [10].

Digestion of food is known to significantly alter hemodynamics [11–13], and may therefore affect MPI, as loading conditions are altered. We have previously reported data from our cohort on the effect of food intake on systolic and diastolic function [14–16]. The purpose of the present study is to evaluate the hypothesis that food intake, in healthy volunteers, may have an effect on myocardial performance index, as it is considered a reflection of the total left ventricular function. To our knowledge this has not been investigated previously.

## Methods

### Study population

Study subjects were 23 healthy volunteers (11 male and 12 female aged 25.6 ± 4.5 years]. No subjects had symptoms or history of cardiovascular disease or any other chronic diseases. None of the subjects were taking cardiovascular medication. Other exclusion criteria were inappropriate acoustic windows and non-sinus rhythm.

### Procedures

The examinations were performed in the morning after fasting overnight. A baseline echocardiographic exam was performed, after which the subjects ingested a standardized meal consisting of 300 g rice pudding (AXA Goda Gröten Risgrynsgröt; Lantmännen AXA, Järna Sweden). A second echocardiographic exam was performed 30 min after, and a third exam 110 min after the meal. The subjects reassumed a supine position between the echocardiographic examinations.

### Echocardiography

After an initial screening examination to rule out cardiac dysfunction, a transthoracic echocardiographic examination was performed in left lateral position, with Sonos 5500 (Philips, Andover, MA, USA) before, 30 min after, and 110 min after the meal, in each instance after a 15 min rest. A single observer performed all echocardiographic measurements three times on separate cardiac cycles, from which the mean value was derived.

The pulsed Doppler parameters were acquired from the apical four-chamber view, with the sample volume for the signal of the mitral inflow velocity pattern at the tip of the mitral leaflets, and the left ventricular outflow velocity was recorded with the sample volume positioned just below the aortic annulus. Ejection time (ET) was measured as the duration of ventricular outflow. Isovolumetric contraction time (ICT) + isovolumetric relaxation time (IRT) were obtained by subtracting ET from the interval between mitral closure to opening (Fig. 1).

### Statistical analysis

Data are presented as mean ± standard deviation (SD). Statistical analyses were performed using Statistica 7.1 (StatSoft Inc, Tulsa, OK, USA). Comparisons between fasting values for ICT, IRT, ET and MPI versus values 30 and 110 min after the ingestion of food, were analyzed for significance with Wilcoxon matched pairs test. Statistical significance was set at a level of *P* <0.05.

## Results

**Fig. 2:**
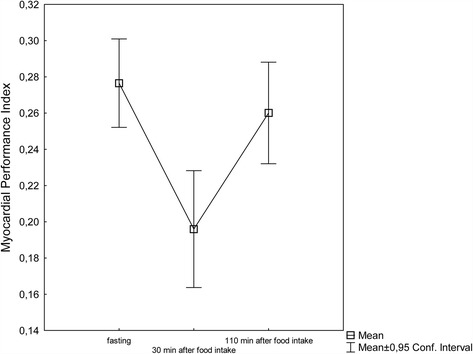
Myocardial performance index (MPI) before (MPI 1), 30 min after (MPI 2, *P* < 0.05), and 110 min after the meal (MPI 3, *P* = 0.15)

All subjects had complete measurements, and no subjects were found to have any cardiac dysfunction. Table 1 summarizes the baseline echocardiography findings. The mean value for MPI decreased significantly 30 min after food intake, and had almost returned to baseline value 110 min after eating. MPI in these young, healthy volunteers decreased from fasting value 0.28 ± 0.06–0.20 ± 0.07 30 min after eating (*P* < 0.05), and was with an index value of 0.26 ± 0.06 almost back to baseline value after 110 min (*P* = 0.15) (Fig. 2). More specifically the sum of ICT + ET + IRT decreased from 0.400 ± 0.024 ms fasting to 0.370 ± 0.030 ms (*P* < 0.001) 30 min after food intake, and to 0.391 ± 0.023 ms (*P* < 0.05) after 110 min. The change in ET, on the other hand, was a slight decrease from fasting value 0.313 ± 0.016 ms to 0.309 ± 0.016 ms (*P* < 0.05) 30 min after eating, and 0.311 ± 0.016 ms (NS) 110 min after food intake. Table 2 summarizes the effect of food intake on blood pressure, heart rate, MPI and selected systolic and diastolic parameters which have been reported earlier [16], but are included for comparison.

**Table 1:** Subjects’ anthropometric characteristics and cardiac dimensions (*n* = 23)

Variable	
Sex (male/female)	11/12
Weight (kg)	68 ± 11
Height (cm)	177 ± 8
BMI (kg/m^2^)	21.7 ± 2.2
BSA (m^2^)	1.8 ± 0.2
LVIDd (mm/m^2^)	26.7 ± 1.9
LVIDs (mm/m^2^)	17.7 ± 1.9
IVSd (mm/m^2^)	5.1 ± 0.4
PWTd (mm/m^2^)	5.0 ± 0.4
LA (mm/m^2^)	18.2 ± 1.9
LVM (g/m^2^)	87.6 ± 18.1

Abbreviations: Indexed for BSA: *LVIDd* left ventricular internal dimension in diastole, *LVIDs* left ventricular internal dimension in systole, *IVSd* interventricular septum thickness in diastole, *PWTd* posterior wall thickness in diastole, *LA* left atrial end-systolic diameter, and *LVM* left ventricular mass, *BSA* Body surface area

Values are mean ± SD

**Table 2:** Description of hemodynamics, blood pressure, heart rate, MPI and and selected systolic and diastolic parameters before, 30 and 110 min after a standardized meal (*n* = 23)

Variable	Fasting	30 min after food intake	110 min after food intake	Percent change fasting versus 30 min (%)
MPI left ventricle	0.28 ± 0.06	0.20 ± 0.07^***^	0.26 ± 0.06	−29
Heart rate (bpm)	60 ± 8	64 ± 10^**^	60 ± 10	7
Systolic BP (mm hg)	103 ± 9	102 ± 10	102 ± 9	−1
Diastolic BP (mm hg)	66 ± 7	58 ± 7^**^	63 ± 6 ^*^	−12
SV (ml)	66 ± 13	79 ± 16^***^	70 ± 13^**^	20
CO (ml/min)	3948 ± 831	5058 ± 1087^***^	4110 ± 756	28
E (cm/s)	81.6 ± 13.2	88.1 ± 14.5^**^	81.0 ± 13.0	8
A (cm/s)	44.3 ± 8.4	51.0 ± 10.6^**^	46.9 ± 7.7	15
E/A	1.9 ± 0.4	1.8 ± 0.4	1.8 ± 0.4	−5
DT (msec)	191 ± 21	162 ± 20^***^	177 ± 17^*^	−15
s’ (septal) (cm/s)	8.3 ± 0.9	9.5 ± 1.2^***^	9.3 ± 1.1^***^	14
s’ (lateral) (cm/s)	12.4 ± 2.2	13.8 ± 2.1^**^	13.0 ± 2.3	11
e’ (septal) (cm/s)	12.6 ± 2.0	13.0 ± 2.2	13.3 ± 2.6	3
e’ (lateral) (cm/s)	19.7 ± 4.7	21.1 ± 4.0	19.2 ± 3.6	7
E/e’ (septal)	6.6 ± 1.3	6.9 ± 1.3	6.3 ± 1.4	5
E/e’ (lateral)	4.3 ± 0.9	4.3 ± 0.8	4.3 ± 0.9	0
E/e’ (average)	5.1 ± 0.9	5.2 ± 0.8	5.0 ± 0.9	2

Abbreviations: *MPI* Myocardial performance index, *BP* Blood pressure, *SV* left ventricular stroke volume, *CO* left ventricular cardiac output, *E* Peak of early diastolic mitral flow velocities, *A* late diastolic mitral flow velocities. *DT* Deceleration time of E-wave, *s’* Pulsed Tissue Doppler imaging velocities: peak systolic diastolic velocities, *e’* Pulsed Tissue Doppler imaging velocities: early diastolic velocities

^*^Indicates significant difference (*P* < 0.05), compared to fasting values

^**^Indicates significant difference (*P* < 0.01), compared to fasting values

^***^Indicates significant difference (*P* < 0.001), compared to fasting values

Values are mean ± SD. Last column shows percent change for the different values, fasting versus 30 min after food intake

## Discussion

This study shows that myocardial performance index is affected by food intake in healthy subjects. Since hemodynamics are known to change postprandially, it is not unreasonable to assume that MPI would be altered accordingly. Although there was a small decrease in ejection time, the main alteration leading to the significant decrease in MPI after eating is the decrease in ICT + IRT. In comparison to our previous findings [14–16] the change in MPI is larger than the changes seen for diastolic parameters, and of the same magnitude as several systolic changes. The exact mechanisms behind the findings in the present investigation are hard to define. Several kinds of postprandial cardiovascular changes have, however, been reported in the literature. It has been suggested that the increase in postprandial cardiac output is the result of increases in bloodflow in the superior mesenteric artery, the heart rate and stroke volume [13]. The correlation between heart rate and MPI has however been found to be insignificant [1] or weak [17]. Physiological changes in the levels of glucose, insulin, glucagon-like peptide 1 (GLP-1) and ghrelin may also influence the activity of the heart [18]. Moreover, it is known that insulin has positive chronotropic and inotropic effects on the heart [19], and the hormone GLP-1 has been shown to improve left ventricular function [20, 21]. The hormone ghrelin has been shown to increase cardiac output (CO) and stroke volume (SV) [22–24]. The ingestion of food has also been shown to decrease the diastolic blood pressure [18]. Considering the changes in hemodynamics with increased cardiac output and altered loading conditions it is not surprising that MPI – and more specifically the isovolumetric contraction and relaxation - changes accordingly.

Myocardial performance index has been used to investigate different populations, such as patients with heart failure [2], acute myocardial infarction [3], heart transplants [4], COPD [25], patients with chronic ischemic cardiomyopathy who have undergone revascularization [26], as well as in healthy young men during high altitude exposure [27]. MPI has also been investigated in relation to age [28].

The time frames between food intake and echocardiographic examinations have, however, not been specified in these studies, suggesting that these were not controlled. While it is difficult to control patients’ food intake in clinical echocardiography, one should be aware of the effect that food intake has on this echocardiographic parameter. The influence of food consumption should be considered in studies, especially when a small sample size is involved.

This investigation was limited by our inability to perform the echocardiographic exams blinded to the state of food intake, because a single observer performed all exams. In an attempt to avoid bias, all exams were stored digitally and the measurements were performed later in random order. The change in MPI had not yet returned to baseline values 110 min after food intake, and in hindsight we would have chosen a longer time period. Moreover, we did not include a control group who did not receive the prepared meal after overnight fasting. The present study shows the effect of food intake only in young, healthy subjects. Additional studies are warranted in older healthy subjects and in patients with various health conditions to determine whether the findings in the present study are reproducible in such populations.

## Conclusions

This study shows that myocardial performance index is affected by food intake in healthy subjects.

## Abbreviations

A: Late diastolic mitral flow velocities
BP: Blood pressure
CO: Left ventricular cardiac output
DT: Deceleration time of E-wave
E: Peak of early diastolic mitral flow velocities
e’: Pulsed Tissue Doppler imaging velocities: early diastolic velocities
ET: Ejection time
GLP-1: Glucagon-like peptide 1
ICT: Isovolumetric contraction time
IRT: Isovolumetric relaxation time
MPI: Myocardial performance index
s’: Pulsed Tissue Doppler imaging velocities: peak systolic diastolic velocities
SD: Standard deviation
SV: Left ventricular stroke volume
